# Induction of N-Ras degradation by flunarizine-mediated autophagy

**DOI:** 10.1038/s41598-018-35237-2

**Published:** 2018-11-16

**Authors:** Ze-Yi Zheng, Jing Li, Fuhai Li, Yanqiao Zhu, Kemi Cui, Stephen T. Wong, Eric C. Chang, Yi-Hua Liao

**Affiliations:** 1 Lester and Sue Smith Breast Center, and Department of Molecular and Cellular Biology, Baylor College of Medicine, Houston, TX 77030 USA; 2 Department of Oncology and Hematology, Hospital (TCM) Affiliated to Southwest Medical University, Luzhou, Sichuan 646000 P. R. China; 3 Department of Systems Medicine and Bioengineering, Houston Methodist Research Institute, Houston, TX 77030 USA; 4 Department of Dermatology, National Taiwan University Hospital and National Taiwan University College of Medicine, Taipei, 10002 Taiwan

## Abstract

Ras GTPases are powerful drivers for tumorigenesis, but directly targeting Ras for treating cancer remains challenging. The growth and transforming activity of the aggressive basal-like breast cancer (BLBC) are driven by N-Ras. To target N-Ras in BLBC, this study screened existing pharmacologically active compounds for the new ability to induce N-Ras degradation, which led to the identification of flunarizine (FLN), previously approved for treating migraine and epilepsy. The FLN-induced N-Ras degradation was not affected by a 26S-proteasome inhibitor. Rather, it was blocked by autophagy inhibitors. Furthermore, N-Ras can be seen co-localized with active autophagosomes upon FLN treatment, suggesting that FLN alters the autophagy pathway to degrade N-Ras. Importantly, FLN treatment recapitulated the effect of *N-RAS* silencing *in vitro* by selectively inhibiting the growth of BLBC cells, but not that of breast cancer cells of other subtypes. In addition, *in vivo* FLN inhibited tumor growth of a BLBC xenograft model. In conclusion, this proof-of-principle study presents evidence that the autophagy pathway can be coerced by small molecule inhibitors, such as FLN, to degrade Ras as a strategy to treat cancer. FLN has low toxicity and should be further investigated to enrich the toolbox of cancer therapeutics.

## Introduction

Humans have three *RAS* genes, *H-*, *N-*, and *K-RAS*. The best known Ras activity for tumorigenesis is to mediate growth factor signaling at the plasma membrane. Oncogenic mutations rendering Ras proteins constitutively active are among the most frequent genetic alterations in human tumors — over 30% of all human tumors contain an oncogenic *RAS* mutation^1^. While *K-RAS* is the most frequently mutated *RAS* gene in cancers overall, *N-RAS* alterations occur in certain cancer types. For example, approximately 20% of melanomas are already known to carry oncogenic *N*-*RAS*^2^. Beside oncogenic *RAS mutations*, we have recently shown that an increased activity of a *wild type* N-Ras is both necessary and sufficient to drive the formation and/or progression of one breast cancer subtype, the “basal-like” breast cancer (BLBC)^3^, which is a sub-group of breast cancer that usually does not express estrogen receptor-α (ER), progesterone receptor (PR), or HER2. BLBC is of great clinical importance because it is aggressive with poor prognosis^4^, and lacks a targeted therapy.

Despite the importance of Ras in cancer, there is no drug yet that specifically targets Ras proteins. The most common “Ras inhibitors” actually target farnesyltransferases (farnesyltransferase inhibitors, FTIs) to reduce general membrane affinity of Ras proteins. However, FTIs can inactivate other prenylated proteins (≥2% of all proteins are prenylated) or cause some Ras proteins to instead undergo geranylgeranylation, thus retaining similar membrane affinity^5^. These and other factors greatly reduce FTI effects on treating Ras-driven cancers^6^.

An alternative strategy to treat Ras-driven cancers is to target the Ras pathway by blocking Ras-effector interaction^7^ or by inhibiting the activity of effector protein kinases, such as B-Raf. The latter approach, in particular, has resulted in many drugs currently in use in the clinic. However, drug resistance to B-Raf inhibitors (e.g., PLX4032/Vemurafenib) can later develop, and one resistance mechanism is to acquire an oncogenic *N-RAS* mutation, whose effectors are unknown^8^. Furthermore, blockade of receptor tyrosine kinases (RTKs) upstream of Ras can also be bypassed by the emergence of oncogenic *K-RAS*^9,10^. Most remarkable, in colon cancer carrying a *BRAF-V600E* mutation, oncogenic *K-RAS* and *N-RAS* are common in relapsed cancers despite single or even combination treatments targeting RTK, Raf, and MEK^11^. Therefore, it seems highly desirable to target Ras proteins themselves in order to shut down all of their oncogenic potential at the root.

Levels of Ras proteins can be controlled post-transcriptionally by proteolysis. Ras proteins can be degraded by the lysozomes^12^ or by the 26S proteasomes, the latter of which are highly relevant to colorectal cancer^13,14^. We therefore hypothesized that it would be possible to target the control of Ras protein levels as a drug development strategy. In this study, we have conducted a screen to re-purpose FDA-approved drugs for the new ability to induce Ras degradation. We have chosen N-Ras as a proof of principle that we can harvest the power of cellular proteolytic pathways to treat Ras-driven cancers, such as BLBC. Our study has identified flunarizine (FLN), classified originally as a Ca^2+^-channel blocker that is used frequently to treat migraine and epilepsy. FLN has not been directly used to treat cancers, but it can enhance sensitivity of cancer cells for some chemotherapy agents^15,16^. Our data show here that FLN can induce an autophagy pathway to selectively degrade N-Ras, thus blocking the growth of BLBC cells *in vitro* and *in vivo*.

## Results

### Screen for compounds that induce reduction in N-Ras protein levels

**Figure 1:**
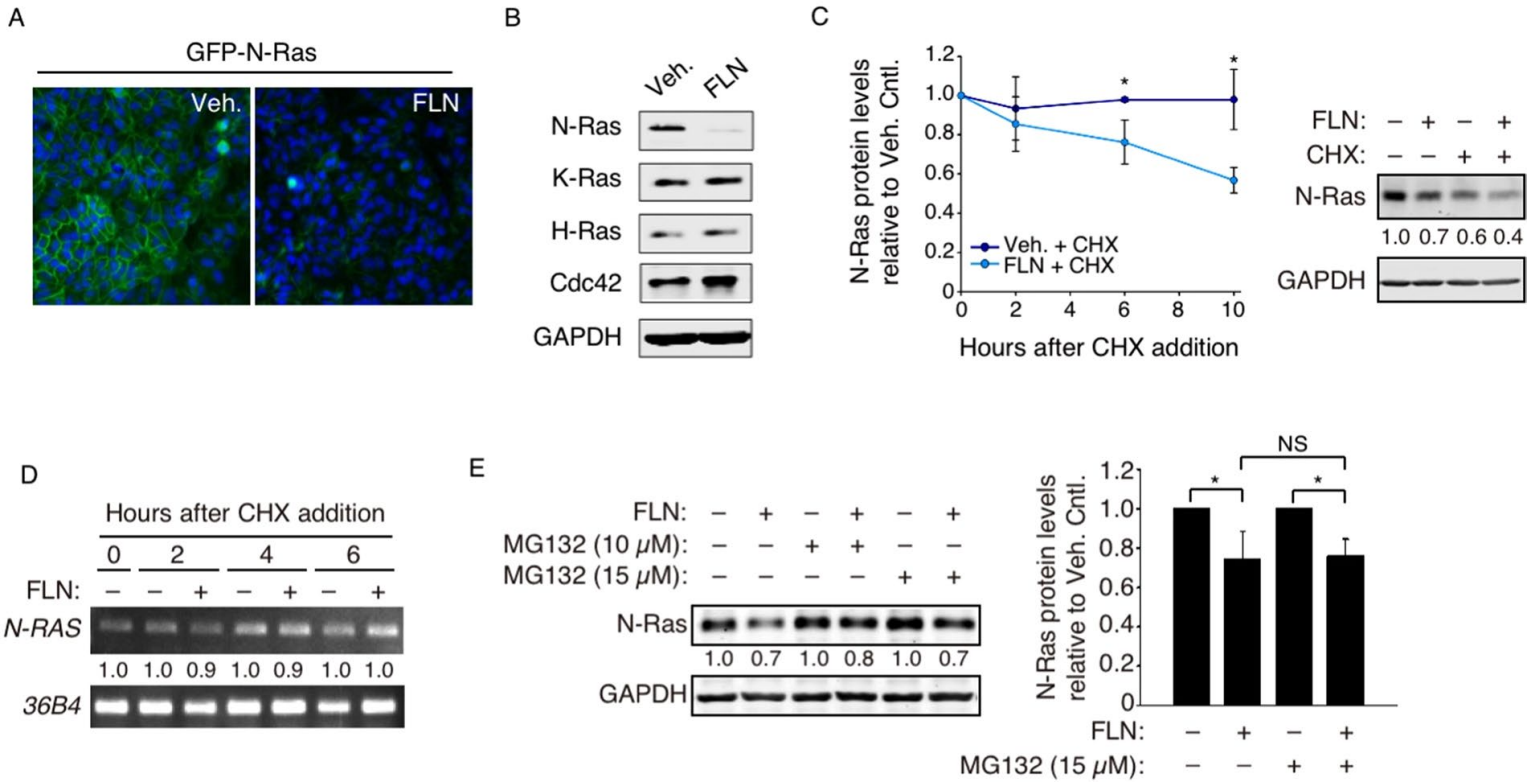
FLN-induced N-Ras degradation is mainly proteasome-independent. (**A**) MDCK cells stably expressing a fluorescently tagged N-Ras protein (GFP-N-Ras, green) were examined by microscopy after FLN treatment, and DAPI (blue) was added to mark the nucleus. (**B**) Western blot analysis of SUM149PT cells treated by FLN (20 µM, 48 hours). H-Ras, K-Ras, Cdc42, and GAPDH were examined as controls. Original full length gels for this and other figures can be found in Supplemental Information. (**C**) SUM149PT cells were pre-treated with vehicle or FLN, and time points were taken and analyzed by Western blot after CHX addition (Left). A representative immunoblot 10 hours post-CHX treatment is shown on the right. Numbers below are GAPDH-normalized N-Ras levels relative to that in the vehicle control. (**D**) *N-RAS* mRNA time points in panel-*C* were also analyzed by semi-quantitative RT-PCR, normalized by levels of control *36B4* mRNA. (**E**) Left, SUM149PT cells treated with indicated drugs for 6 hours were analyzed by Western blot. The numbers show N-Ras levels relative to those in cells not treated by FLN. Data from 3 separate experiments are quantified on the right.

MDCK cells stably expressing GFP-tagged N-Ras (Fig. 1A) were screened by microscopy for compounds that can greatly reduce GFP-N-Ras levels. MDCK cells were chosen for the screen because of the cobblestone-like cell morphology, which makes it easier to visualize plasma membrane proteins. The compound library of choice was LOPAC, which contains a large collection of pharmacologically active compounds that target a wide range of cellular processes. Compounds that reduced GFP signals by ≥50% were further tested by Western blot in several BLBC cells, and FLN is the only identified compound that can selectively and reproducibly reduce endogenous N-Ras protein levels, without significantly affecting levels of control proteins such as H-Ras, K-Ras, Cdc42, and GAPDH. An example of such an experiment with SUM149PT cells is shown in Fig. 1B.

### FLN promotes N-Ras degradation

The BLBC SUM149PT cells were further examined to determine whether the decrease in N-Ras levels was due to protein degradation or mRNA reduction. When cycloheximide (CHX) was added after FLN, N-Ras protein levels declined over time, as measured by Western blot, indicating shortening of protein half-life (Fig. 1C). In contrast, no substantial decline of *N-RAS* mRNA levels in response to FLN was observed, as measured by semiquantitative RT-PCR (Fig. 1D). These data strongly suggest that the observed decrease in N-Ras levels was mainly due to increased protein degradation. To assess whether FLN-induced N-Ras degradation was catalyzed by the 26S-proteasome, the proteasome inhibitor MG132 was added to FLN-treated SUM149PT cells, and the data showed that MG132 did not substantially inhibit FLN-induced N-Ras degradation, suggesting that this activity was primarily independent of the 26S proteasome (Fig. 1E).

### FLN induces N-Ras recruitment into the autophagosome

**Figure 2:**
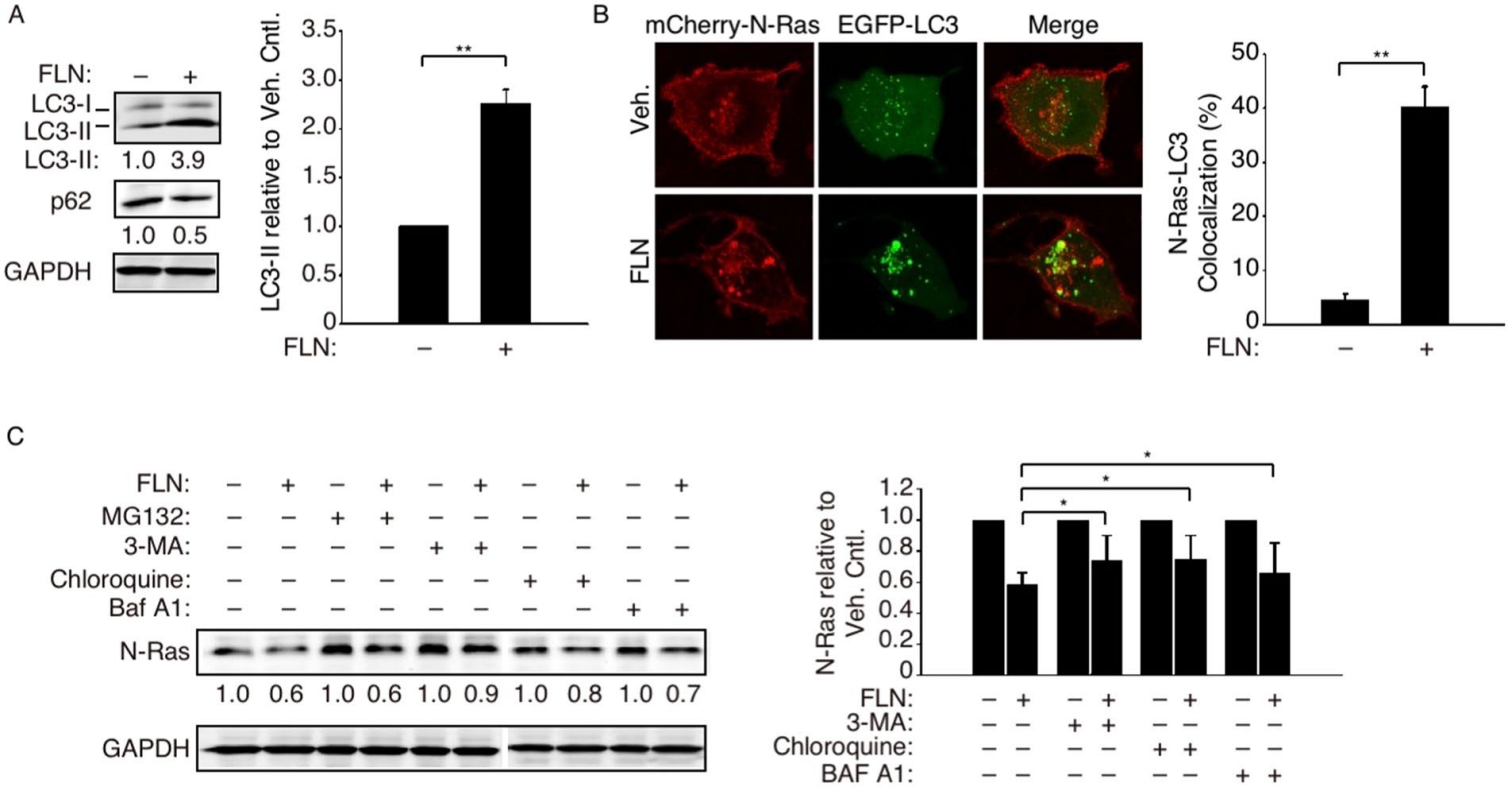
FLN enhanced autophagic influx and N-Ras translocation to autophagosomes. (**A**) Left, Western blot analysis of LC3-II and p62/SQSTM-1 in SUM149PT cells treated with 20 µM FLN for 24 h. Numbers below show protein levels relative to that in the vehicle control cells. Right, quantification of relative LC3-II levels from three independent experiments. (**B**) Cells co-transfected with mCherry-N-Ras and EGFP-LC3 were imaged by confocal microscopy after FLN treatment (left). Percent co-localization (see Methods) was quantified on the right (n = 6 cells). (**C**) Left, SUM149PT cells were first treated with FLN as in panel-A, followed by autophagy inhibitors 3-MA (5 mM), chloroquine (20 µM) or bafilomycin A1 (20 nM) for another 24 hours. The proteasome inhibitor MG132 (15 µM) was included as a negative control to show unabated N-Ras reduction. The cells were then harvested and analyzed by Western blot. The numbers show N-Ras levels relative to those in cells not treated by FLN. Data from three independent experiments were quantified on the right.

By microscopy, we noted an increase in cellular vacuolization that resembles activation of autophagy after FLN treatment. Despite being known for its anti-Ca^2+^ channel activity, FLN has also been reported to induce autophagy-like activity in glioblastoma cells^17^. SQSTM1/p62 degradation is a marker for active autophagy^18^, and we observed a decrease in p62 levels by FLN in SUM149PT cells (Fig. 2A). Furthermore, the conversion of LC3 (microtubule-associated protein 1 A/1B-light chain 3) to the faster-migrating LC3-II marks active autophagosomes where this process takes place^19^, and we found such an increase in LC3-II in the FLN-treated SUM149PT cells. These results suggest that FLN also activates LC3-containing autophagosomes in BLBC cells. To assess whether N-Ras is recruited into these autophagosomes upon FLN treatment, mCherry-N-Ras and EGFP-LC3 were co-expressed and analyzed by confocal microscopy. Our data showed that EGFP-LC3 formed a pronounced coarse punctate pattern indicative of strong autophagy activation (Fig. 2B). Likewise, instead of mostly being concentrated at the plasma membrane and Golgi, FLN-induced mCherry-N-Ras also accumulated in such structures, leading to substantial co-localization in the coarse punctate pattern with EGFP-LC3 (Fig. 2B).

### FLN-induced N-Ras degradation requires a functional autophagy pathway

To assess whether FLN-induced N-Ras degradation is functionally dependent on autophagy, SUM149PT cells were treated with chloroquine or bafilomycin A1, which can prevent autophagosome-lysosome fusion. Our data showed that these drugs partially restored N-Ras levels (Fig. 2C). Partial restoration of N-Ras levels was also achieved by 3-methyladenine (3-MA; Fig. 2C), which prevents autophagosome formation^20^. All together, these data suggest that FLN controls N-Ras protein levels mainly by activating an LC-3 dependent autophagy pathway, leading to N-Ras engulfment and degradation.

### FLN inhibits growth and transforming activity of BLBC cells with selectivity

**Figure 3:**
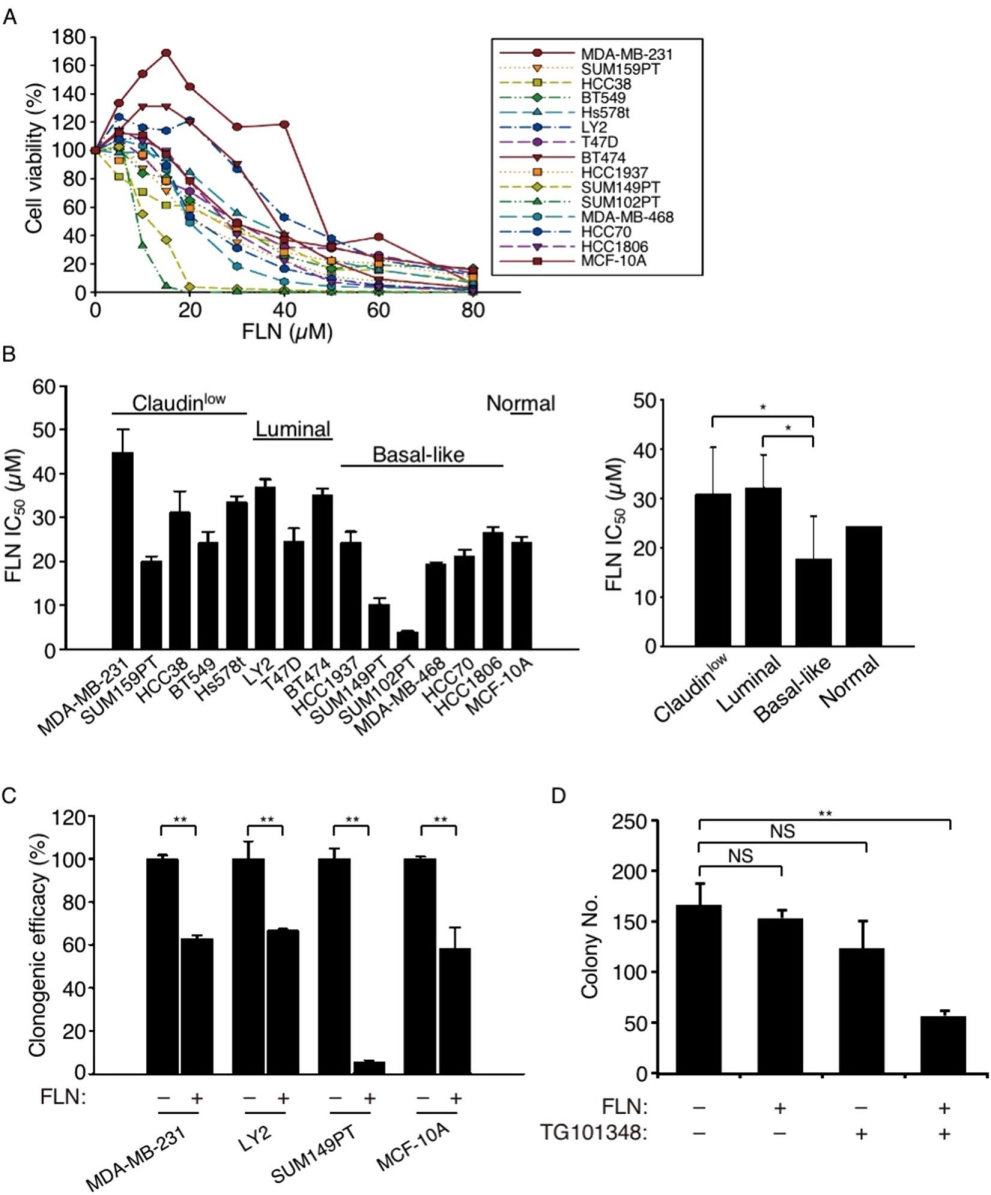
FLN inhibits the growth of BLBC cells more efficiently than of other breast cancer subtypes. (**A**) Indicated breast cell lines were treated with different concentrations of FLN for 48 hours before the cell viability was measured by MTS assay. (**B**) The IC_50_s of FLN for each cell line were calculated from the growth experiment in panel-A (left) and grouped and quantified based on subtypes (right). (**C**) MDA-MB-231 (claudin-low), LY2 (luminal), SUM149PT (basal-like), and the untransformed mammary epithelial cells MCF-10A were treated with FLN 20 µM for 6 days for colony formation. n = 4 experiments. (**D**) SUM102PT cells were treated with 0.5 µM FLN and/or 0.1 µM TG101348 in soft-agar for 21 days and stained by MTT.

N-Ras has been shown to be both necessary and sufficient for the growth and transforming activity of BLBC cells^3^. For example, *N-RAS*-silencing does not substantially affect the growth of un-transformed breast epithelial cells (e.g., MCF-10A cells) or that of luminal and claudin-low breast cancer cells^3^. We thus measured growth of a panel of breast cancer cells, as well as the untransformed MCF-10A cells, at various concentrations of FLN (Fig. 3A). Based on IC_50_s of FLN, there is evidence that BLBC cells are more sensitive to FLN (Fig. 3B). Among BLBC cells, SUM149PT and SUM102PT cells were more sensitive to FLN, which agrees with our previous findings that they were also more sensitive to N-Ras inhibition by shRNA^3^. In addition, we selected one cell line each from 3 breast cancer subtypes, as well as MCF-10A cells, and performed clonogenic assays at a concentration of FLN close to the IC_50_ of BLBC cells. Once again, FLN recapitulated the effects of *N-RAS* silencing^3^ in that the BLBC SUM149PT cells were the most sensitive to FLN, as compared to MCF-10A, the claudin-low MDA-MB-231, and the luminal LY2 cells (Fig. 3C).

Instead of activating the conventional Raf effector pathway, N-Ras in BLBC cells has been shown to act through JAK2, and colony formation efficiency of these cells in soft agar can be inhibited by a JAK2 inhibitor, TG101348^3^. In this study, we examined sub-IC_50_ concentrations of TG101348 and FLN and found that when used alone they had little effect on soft agar colony formation by another BLBC cell line, SUM102PT (Fig. 3D); however, when the two drugs were combined, colony formation was greatly inhibited (Fig. 3D). These results suggest that FLN, like *N-RAS* silencing, can efficiently inhibit growth and transforming activity of BLBC cells, and this inhibition can be further enhanced by combining with drugs targeting additional components in this N-Ras pathway.

### Tumor growth inhibition by FLN in a xenograft model of BLBC

**Figure 4:**
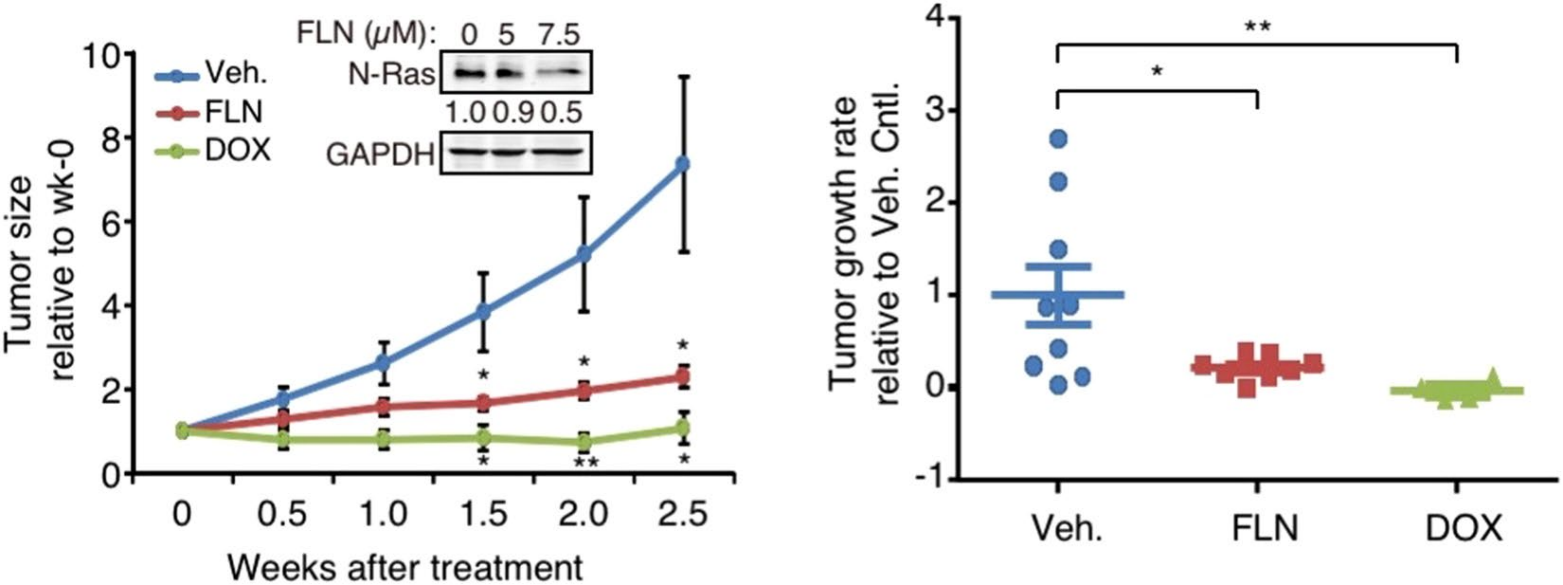
FLN inhibits tumor growth of a BLBC xenograft model. SUM102PT cells were transplanted into the mammary glands of nude mice. When tumors became palpable, the mice bearing the tumors were randomized to be treated with either DOX or FLN. Tumor sizes relative to those at week-0 were plotted on the left. n = 9, 8, and 4 mice in vehicle, FLN, and DOX-treated groups respectively. Inset: FLN-induced N-Ras reduction was validated in SUM102PT cells. Tumor growth rate of each mouse was calculated and plotted on the right. Data were presented as Mean ± s.e.m. The pair-wise comparison was between each treatment group and the vehicle control.

To assess whether FLN can inhibit growth of BLBC tumors *in vivo*, the SUM102PT xenograft mouse model was chosen because its growth is strongly N-Ras-dependent^3^. Our data showed that when FLN was added at levels comparable to those used in humans^21,22^, tumor growth was efficiently inhibited, mimicking the effect of the doxycycline (DOX)-inducible *N-RAS* silencing (Fig. 4).

## Discussion

Despite the importance of Ras proteins in driving tumor formation, directly targeting Ras for cancer treatment remains mostly unsuccessful. While Ras proteins are generally considered to be stable proteins, they are evidently under the surveillance of proteolytic pathways. This study presents evidence that N-Ras can be selectively degraded by the autophagy pathway in the presence of FLN.

While known primarily as a Ca^2+^-channel blocker, FLN has been shown to increase autophagy activity in other cell types. Therefore, autophagy activation is likely to be another intrinsic activity of FLN that is not restricted to a specific cell type. However, when FLN is given to cancer cells, such as BLBC cells, whose growth is strongly driven by N-Ras, selective inhibition of cell and tumor growth of these cells can be achieved. Because FLN has low toxicity, it may be more easily combined with other drugs, such as JAK2 inhibitors, to further enhance treatment efficacy. JAK2 inhibitors are currently in clinical trial for treating ER-PR-HER2 triple-negative breast cancer^23^, which includes BLBC.

While autophagy was first discovered as a non-selective process to recycle building blocks for cells that are starving, increasing evidence suggests that the autophagy pathways can degrade a subset of proteins or even whole organelles with selectivity^24^. Indeed, FLN selectively induces N-Ras to be degraded while K-Ras and several other proteins are not readily degraded by this process. Future studies are needed to define how N-Ras is selected for degradation by autophagy in the presence of FLN.

## Materials and Methods

All general methods were performed in accordance with the relevant guidelines and regulations, and animal work in particular was conducted in accordance with a protocol approved by the Baylor College of Medicine Institutional Animal Care and Use Committee.

### The drug screen

The Library of Pharmacologically Active Compounds library (LOPAC^1280^, Sigma-Aldrich) contains 1,280 compounds with well-known pharmacological properties that target a wide range of cellular activities. Many compounds in this library have been approved for use in the clinic, which greatly increases the possibility of re-purposing relatively non-toxic and cheap drugs for new treatment of cancers. Canine kidney epithelial MDCK cells were transduced to stably express a GFP-tagged N-Ras (GFP-N-Ras). These cells were seeded in 96-well plates to which 10 µM of each compound was added. The cells were incubated with the drug for 2 days before being analyzed by microscopy, for which we have developed an algorithm to identify individual GFP^+^ cells in order to calculate GFP levels in the cell in an automated fashion^25^.

### Cell culture

The breast cancer cell lines have been described previously^3^, and transfection was performed using Lipofectamine 2000 (Invitrogen).

### Antibodies and chemicals

The antibodies against N-Ras (F155), K-Ras (F234), H-Ras (F235), Cdc42(P1) and GAPDH (6C5) were from Santa Cruz Biotechnology. Antibodies against LC3B and p62/SQSTM1 were from Novus Biologicals (NB100–2220) and GeneTex (GTX100685), respectively. Flunarizine dihydrochloride (FLN) used *in vitro*, cycloheximide (CHX), MG132, bafilomycin A1, chloroquine diphosphate salt, and 3-methyladenine were all from Sigma-Aldrich. FLN used *in vivo* was from Medchemexpress.

### Plasmids

Cloning of pCL/mCherry-N-Ras was as described previously^26^. The EGFP-LC3 expression plasmid was a kind gift from Prof. An-Li Cheng, National Taiwan University Hospital.

### Western blotting

The cell lysates were generally prepared in RIPA buffer. Proteins were separated by SDS-PAGE and transferred to nitrocellulose membrane (Bio-Rad). After blocking in 5% non-fat milk in PBS, the membrane was subjected to immunoblotting with primary antibodies. The fluorescein-conjugated secondary antibodies were from Li-COR Biosciences, and the protein levels were quantified by an Odyssey infrared imaging system (Li-COR Biosciences).

### Protein turnover assay and semiquantitative RT-PCR

For cycloheximide (CHX)-chase assays, SUM149PT cells were pre-treated with DMSO (vehicle control) or 20 µM FLN for 14 hours before CHX was added at 50 µM to inhibit further protein synthesis. Time points after CHX addition were harvested for Western blot analysis. Total RNA was also extracted from the cells after CHX addition using the RNeasy Mini Kit (Qiagen), and the cDNAs were generated using the SuperScript First-Strand Synthesis System (Invitrogen). The cycle number was adjusted to allow detection within the linear range of product amplification. The forward and reverse primers for *N-RAS* gene were (5′ to 3′): CACCATGACTGAGTACAAACTG and TTACATCACCACACATGGCAA. The forward and reverse primers for the internal control *36B4* gene were (5′ to 3′): GATTGGCTACCCAACTGTTGCA and CAGGGGCAGCAGCCACAAAGGC.

To inhibit 26S proteasomes, cells were first treated with FLN for 18 hours, and then MG132 was added at final concentrations of 10 or 15 µM for 6 hours. Cell lysates were harvested for Western blot.

### Colocalization analysis by confocal microscopy

HT1080 cells were chosen for this experiment because of their high transfection efficiency. These cells were seeded in glass-bottom plates (MatTek) and co-transfected to express mCherry-N-Ras and EGFP-LC3. One day later, FLN (40 µM) was added for another 24 hours. Live cell imaging was performed on a Leica TCS SP5 confocal microscope with a 63×/1.4 oil objective. To detect GFP and mCherry, 30% of available argon laser power and 80% of available He-Ne laser power were used, respectively. Images were analyzed as described previously^27^ using the LAS AF software (Leica) to calculate percent colocalization between LC3-EGFP and N-Ras-mCherry in a cell.

### IC_50_ measurement

Cells were seeded onto 96-well plates in triplicate before being treated with varying concentrations of FLN for 48 hours. The number of viable cells was measured by CellTiter 96 AQueous One Solution Cell Proliferation Assay kit (Promega). The inhibition curves and the IC_50_ were generated by Prism 6 (GraphPad Software).

### Clonogenic assay

Cells were plated onto a 6-well plate (2,000 cells/well) and treated with either DMSO or FLN for 6 days. Fresh media (with or without FLN) were changed every two days. Colonies were stained with 0.3% crystal violet and scored.

### Soft agar colony formation assay

This was generally conducted as described previously^28^. Briefly, 2 × 10^4^ SUM102PT cells expressing inducible *N-RAS* shRNA^3^ mixed with equal number of human mammary fibroblasts (HMFs) were seeded onto 6-well plates in triplicate. To treat the cells with the drug, 1 ml drug-containing medium was added, which was replaced twice weekly. The cells in soft agar were cultured for 21 days before MTT staining.

### Animal experiments

SUM102PT cells were co-transplanted with HMFs into the mammary fat pads of nude mice as described previously^3^. When average tumor volume reached ≈200 mm^3^ (week-0), the mice were randomized into different treatment groups. DOX was added in the drinking water at 0.2 mg/ml to silence *N-RAS* expression. FLN was injected intraperitoneally daily at 40 mg/kg body weight. The volumes of each tumor were normalized to those at week-0 and plotted over time. The slope of the linear regressed curve of each tumor was defined as the growth rate.

### Statistics

All reported data were presented as Mean ± s.d. unless otherwise indicated. All reported *P* values were calculated by two-sided Student’s t-test. *P < 0.05; **P < 0.01; NS, Not Significant (*P* ≥ 0.05).

## Electronic supplementary material

Supplemental information
