# Supplementary material for: Induction of N-Ras degradation by flunarizine-mediated autophagy
Source: Sci Rep. 2018 Nov 16;8:16932. doi: 10.1038/s41598-018-35237-2 (PMC6240051; doi:10.1038/s41598-018-35237-2)

Supplemental information

Induction of N-Ras degradation by flunarizine-mediated autophagy

Running title: Degrading N-Ras as a cancer therapy

Ze-Yi Zheng, Jing Li, Fuhai Li, Yanqiao Zhu, Kemi Cui, Stephen T. Wong, Eric C. Chang, and Yi-Hua Liao

Full length gels used in the main figures

Fig. 1B

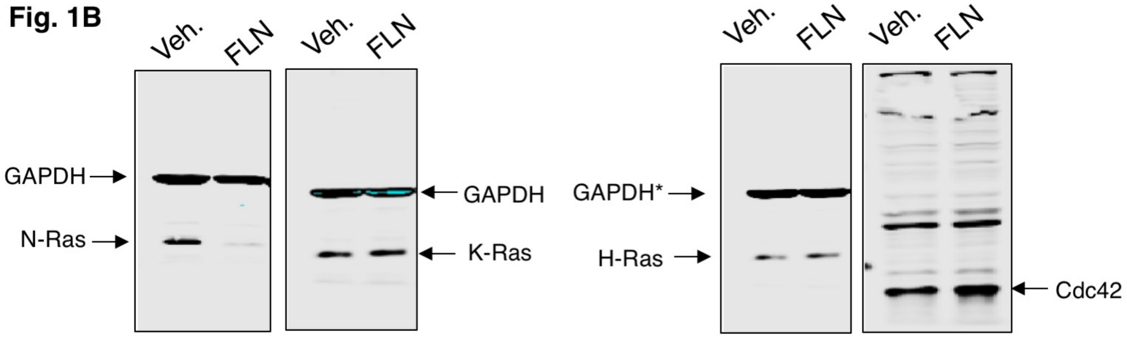

Fig. 1C

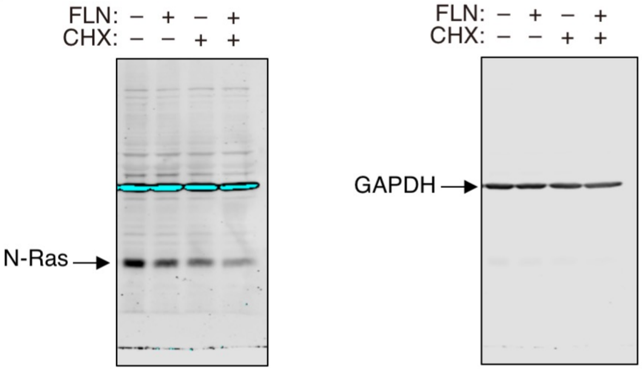

Fig. 1D

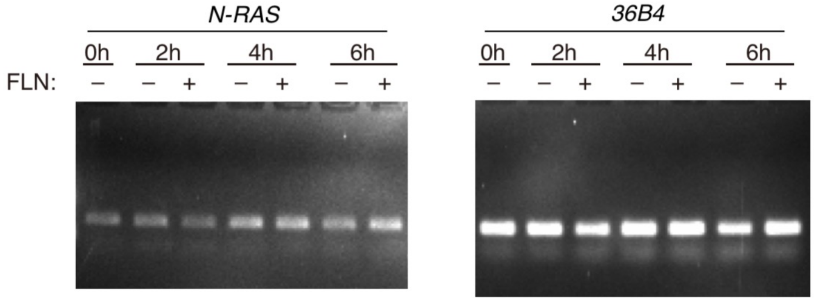

Fig. 1E

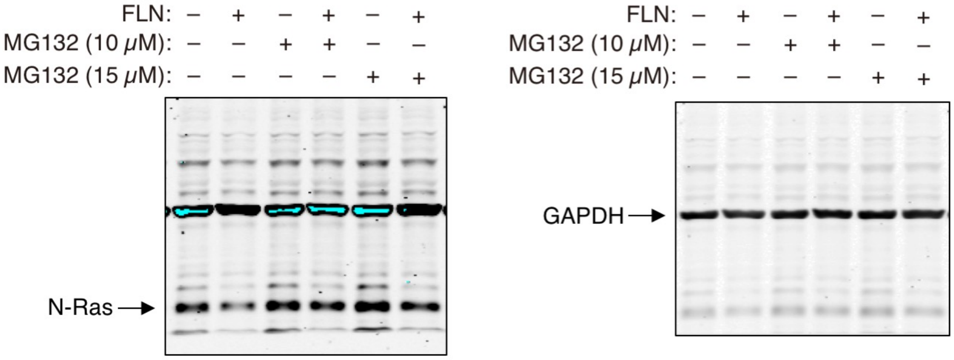

Fig. 2A

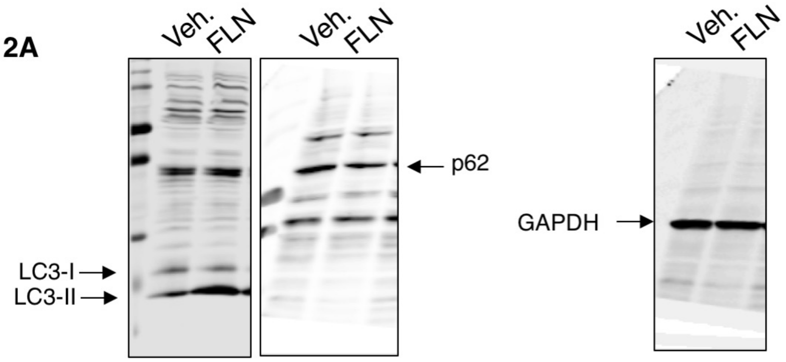

Fig. 2C

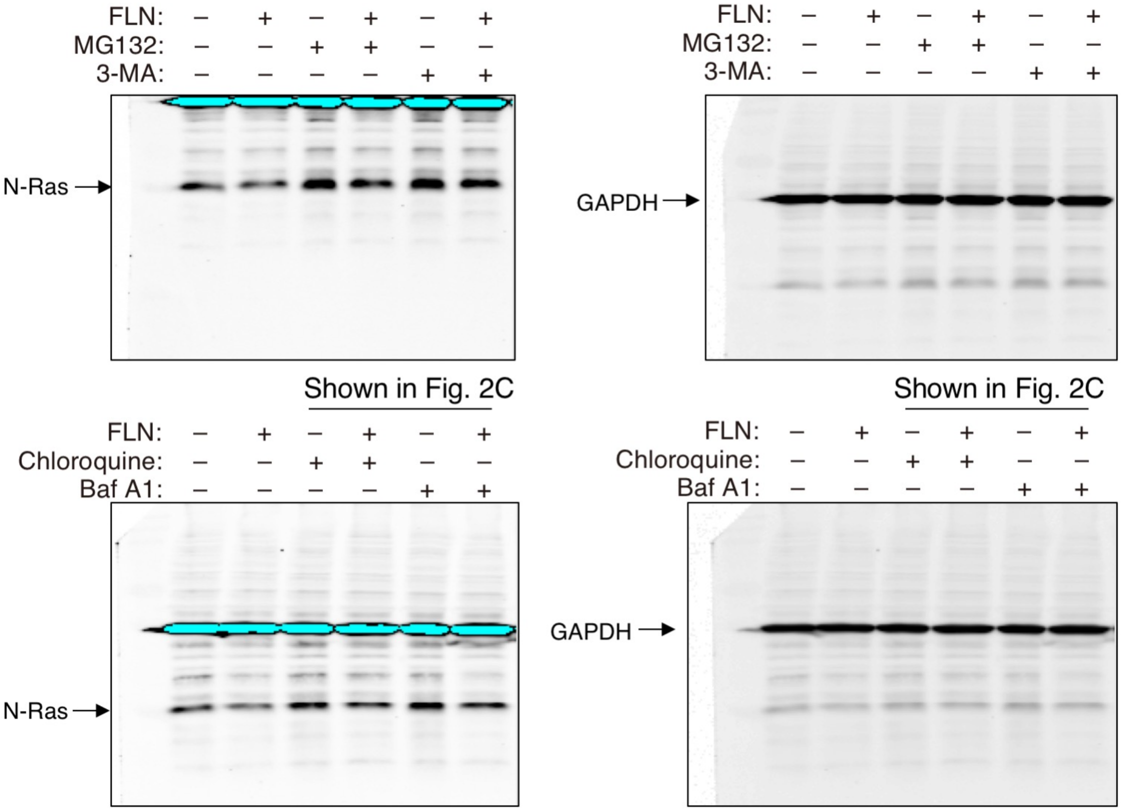

Fig. 4

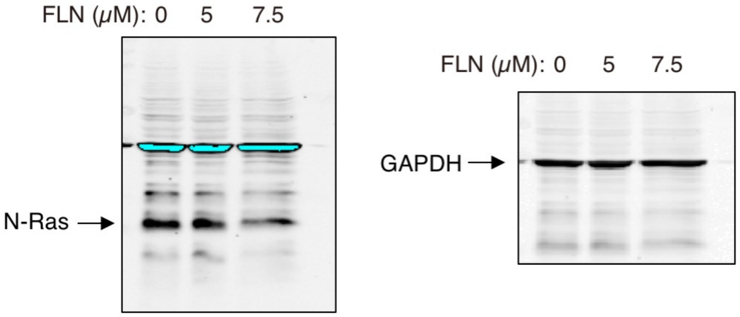

Supplement: Supplementary file 1 — Supplemental information [file 41598_2018_35237_MOESM1_ESM.pdf]
